# Surface sensitivity of atomic-resolution secondary electron imaging

**DOI:** 10.1093/jmicro/dfae041

**Published:** 2024-09-20

**Authors:** Koh Saitoh, Teppei Oyobe, Keisuke Igarashi, Takeshi Sato, Hiroaki Matsumoto, Hiromi Inada, Takahiko Endo, Yasumitsu Miyata, Rei Usami, Taishi Takenobu

**Affiliations:** Institute of Materials and Systems for Sustainability, Nagoya University, Furo-cho, Chikusa-ku, Nagoya 464-8603, Japan; Department of Applied Physics, Graduate School of Engineering, Nagoya University, Furo-cho, Chikusa-ku, Nagoya 464-8603, Japan; Core Technology & Solutions Business Group, Hitachi High-Tech Corporation, 882 Ichige, Hitachinaka, Ibaraki 312-8504, Japan; Core Technology & Solutions Business Group, Hitachi High-Tech Corporation, 882 Ichige, Hitachinaka, Ibaraki 312-8504, Japan; Core Technology & Solutions Business Group, Hitachi High-Tech Corporation, 882 Ichige, Hitachinaka, Ibaraki 312-8504, Japan; Core Technology & Solutions Business Group, Hitachi High-Tech Corporation, 882 Ichige, Hitachinaka, Ibaraki 312-8504, Japan; Department of Physics, Tokyo Metropolitan University, Hachioji, Tokyo 192-0397, Japan; Department of Physics, Tokyo Metropolitan University, Hachioji, Tokyo 192-0397, Japan; Department of Applied Physics, Graduate School of Engineering, Nagoya University, Furo-cho, Chikusa-ku, Nagoya 464-8603, Japan; Department of Applied Physics, Graduate School of Engineering, Nagoya University, Furo-cho, Chikusa-ku, Nagoya 464-8603, Japan

**Keywords:** secondary electron imaging, atomic-resolution scanning electron microscopy, surface sensitivity, atomic layer materials

## Abstract

The surface sensitivity of high-resolution secondary electron (SE) imaging is examined using twisted bilayers of MoS_2_ stacked at an angle of 30°. High-resolution SE images of the twisted bilayer MoS_2_ show a honeycomb structure composed of Mo and S atoms, elucidating the monolayer structure of MoS_2_. Simultaneously captured annular dark-field scanning transmission electron microscope images from the same region show the projected structure of the two layers. That is, the SE images from the bilayer MoS_2_ selectively visualize the surface monolayer. It is noted that the SE yields from the surface monolayer are approximately three times higher than those from the second monolayer, likely attributable to attenuation when SEs emitted from the second layer traverse the surface layer. The surface sensitivity of high-resolution SE imaging is examined using twisted bilayers of MoS_2_ stacked at an angle of 30°. It was found that the SE images of the MoS_2_ bilayer visualize the surface monolayer approximately three times more intensely than the second monolayer.

## Teaser

The surface sensitivity of high-resolution secondary electron (SE) imaging is examined using twisted bilayers of MoS2 stacked at an angle of 30-degree. It was found that the SE images of the MoS2 bilayer visualize the surface monolayer approximately three times more intensely than the second monolayer.

## Introduction

Recent advancements in electron microscopy, such as the correction of spherical aberrations, have significantly enhanced the spatial resolution for observing not only the projected structure of samples using transmission electrons but also surface structures utilizing secondary electrons (SEs) emitted from the sample. The spatial resolution achieved in SE imaging with the latest instrumentation has reached a level that enables the observation of individual uranium atoms dispersed on films and the atomic arrangement in crystals [1, 2].

One of the challenging studies of atom-resolved SE imaging is the observation of surface reconstruction, which was first reported by Ciston *et al*. [3]. They demonstrated that a 2 × 6 reconstruction superstructure of a SrTiO_3_ ($0{\ }0{\ }1$) surface could be extracted from slight differences between SE images of the 1 × 1 fundamental structure and the 2 × 6 superlattice structure. This finding underscored the potential of SE signals to detect surface structures uniquely formed only on the topmost layer of materials with high spatial resolution.

Most SEs generated in samples are generally considered to have an escape depth on the order of a nanometer [4]. The escape depth of SEs is critical for observing surface structures, as it determines the depth limit for obtaining structural information of the specimen surface via SE signals. Recently, Cheng reported, based on an accurate simulation of atomic-resolution SE imaging using the quantum trajectory Monte Carlo method, that a depth difference of 0.2 nm could be identified in a silicon [$1{\ }1{\ }0$] crystal [5]. In this study, we investigate the depth resolution of SE imaging using MoS_2_ bilayers, the thinnest system composed of a surface layer and substrate. The MoS_2_ bilayer is twisted to differentiate between the surface layer and the second layer based on their orientations. Furthermore, we evaluated the spatial resolution and surface sensitivity of the SE images.

## Experimental

Monolayers of MoS_2_ were grown by the chemical vapor deposition (CVD) method on an SiO_2_ thin film formed on a silicon substrate [6] and were transferred by the polymer-assisted dry-transfer method [7, 8]. The monolayers were subsequently coated with an Elvacite polymer acrylic resin (Mitsubishi Chemical Group) using 2D Heterostructure Transfer System (HQ Graphene) and were heated to 70℃ for 5 min to improve adhesion between the monolayer and the polymer. Then, the monolayer was detached from the substrate at 40℃. The detached layer was brought in contact with a Quantifoil holey carbon support film mounted on a transmission electron microscopy (TEM) grid (Quantifoil Micro Tools GmbH) at 120°C to strengthen the adhesion between the monolayer and the grid. The obtained sample was rinsed in isopropyl alcohol and acetone for electron microscopy. Another monolayer was picked up from the substrate and stacked on the monolayer on the grid to synthesize 30°-rotated bilayers of MoS_2_. Since the CVD-grown monolayer had an equilateral triangle shape, the stacking angle of bilayer MoS_2_ was controlled by the relative angle between the edges of the two triangles. The TEM grid with the twisted bilayers was also rinsed in isopropyl alcohol and acetone for electron microscopy.

Atom-resolved SE and annular dark-field scanning transmission electron microscope (ADF-STEM) imaging were carried out using a Hitachi High-Tech HF-5000, equipped with a cold field-emission gun operated at an acceleration voltage of 200 kV. The microscope is equipped with an Everhart–Thornley (ET) SE detector biased at +10 kV. The arrangement of the SE detector in the instrument is the same as that used in the paper by Zhu *et al*. [1] and Inada *et al*. [2]: the SE detector is located above the upper pole of the objective lens pole-piece and the SEs emitted from the sample pass through a hole in the upper pole of the objective lens and are collected in the ET detector. This detector is positioned where the backscattered electrons from the sample are not directly introduced, and the ratio of the backscattered electron signal to the SE signal has been found to be <0.002% [9]. The voltages applied to the sample were set to zero. The convergence semi-angle of the incident beam was set to 23 mrad, and the electron beam current was set to 50 pA. The ADF detector was configured to capture electrons scattered at angles ranging from 40 to 200 mrad. SE and ADF signals were simultaneously acquired with a pixel size of 1024 × 1024 and a dwell time of 19 µs.

The prepared MoS_2_ sample showed polymeric acrylic resin remaining on the surface in a reticulate pattern in the SE image at low magnification. Sample areas with residual acrylic resin on the surface are observed brighter in the SE image than areas without residual resin. The contrast of the MoS_2_ structure image is lower in the areas with residual resin. Atomic-resolution SE and ADF-STEM images of MoS_2_ monolayer and bilayer were acquired by selecting the area where the atomic arrangements of MoS_2_ were most clearly observed free from the background caused by the residual resin. The SE signal was calibrated by setting the SE image intensity from the no-specimen region (i.e. through a vacuum region) to zero. The readout signal from the ET detector is assumed to be linear to the SE yield, since the SE yields in this study are relatively low. Furthermore, ADF-STEM image simulation was conducted using the multislice program xHREM (HREM Research Inc.) with STEM extension, using the same optical parameters as those utilized in the experimental setup.

## Results and discussion

**Fig. 1.**
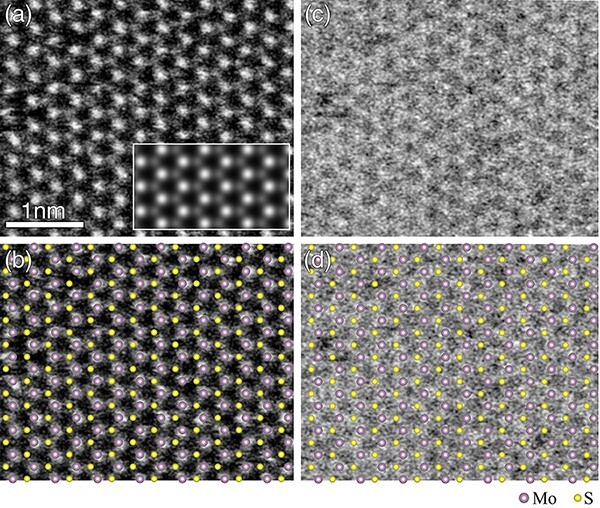
(a) ADF-STEM image of a monolayer of MoS_2_ taken simultaneously at an incidence in the [$0{\ }0{\ }0{\ }1$] orientation. The image shows a good agreement with the simulated image in the lower right inset. (b) Atomic arrangement of the monolayer of MoS_2_ overlaid onto the same images as in (a). The large and small spheres represent Mo and S atoms, respectively. (c) SE image of the monolayer of MoS_2_ taken simultaneously with the ADF-STEM image of (a). (d) Atomic arrangement of the monolayer of MoS_2_ overlaid onto (c).

Figure 1a shows an ADF-STEM image of a monolayer of MoS_2_ taken at an incidence in the [$0{\ }0{\ }0{\ }1$] orientation. Figure 1b shows an atomic arrangement of the monolayer MoS_2_ projected in the [$0{\ }0{\ }0{\ }1$] orientation overlaid onto the ADF-STEM image in Fig. 1a. In this composite image, large spheres represent Mo atoms, while small spheres represent S atoms. It is confirmed that the ADF-STEM image visualizes Mo and S atoms separately and distinguishes Mo and S atoms by the *Z*^2^ contrast (Mo = 42 and S = 16). Some of the atom positions appear displaced or missing from their regular positions, likely due to radiation damage caused by the high-energy electron beam of 200 keV. The simulated ADF-STEM image of an MoS_2_ monolayer, shown in the lower right inset of Fig. 1a, closely matches the experimental ADF-STEM image.

Figure 1c shows an SE image of the monolayer MoS_2_, acquired simultaneously with the ADF-STEM image in Fig. 1a. A Gaussian blur filter with a full-width at half-maximum of 1 pixel has been applied to the image to reduce noise. Figure 1d shows the atomic arrangement of the monolayer projected in the [$0{\ }0{\ }0{\ }1$] orientation overlaid onto the SE image in Fig. 1c. The SE image appears noisier than the ADF-STEM image mainly due to the lower SE yield compared to electrons scattered into the ADF detector. However, the SE image does show signals at positions corresponding to Mo and S atoms, forming the six-membered rings characteristic of the MoS_2_ structure. Mo and S atoms are not distinctly resolved in the SE image, indicating that the inelastic scattering potential associated with SE emission is more delocalized than the elastic scattering potential of ADF-STEM imaging [4, 7]. The elemental contrast in the SE image is almost absent, in contrast to the ADF-STEM image, suggesting that the observed signal does not arise from backscattered electrons following Rutherford scattering proportional to the *Z*^2^, but rather from SEs emitted by the illuminated atoms and their surrounding regions.

**Fig. 2.**
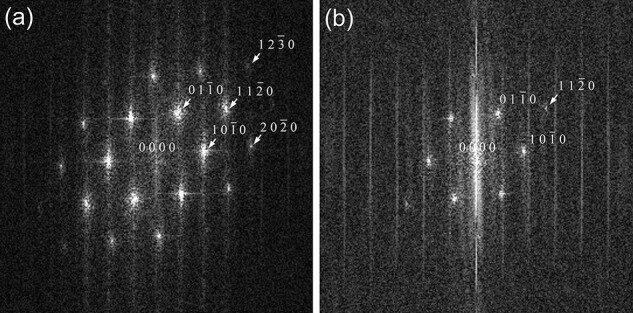
FFT patterns of the ADF-STEM (a) and SE (b) images of the monolayer of MoS_2_ acquired at an incidence in the [$0{\ }0{\ }0{\ }1$] orientation. The outermost peaks of the FFT patterns in (a) and (b) correspond to the ($1{\ }2{\ }\bar 3{\ }0$) plane with a lattice spacing of 0.103 nm and the ($1{\ }1{\ }\bar 2{\ }0$) plane with 0.158 nm, respectively.

Figure 2a and b shows fast Fourier transform (FFT) patterns of the ADF-STEM and SE images, respectively. Both FFT patterns show a 6-fold symmetry. The outermost peaks of the FFT patterns, from which the spatial resolution of the images can be roughly estimated, correspond to the ($1{\ }2{\ }\bar 3{\ }0$) plane with a lattice spacing of 0.103 nm and the ($1{\ }1{\ }\bar 2{\ }0$) plane with 0.158 nm, respectively. One reason for the difference in spatial resolution between the ADF-STEM and SE images may be attributed to the difference in their scattering potentials, as discussed earlier. Since inelastic scattering accompanied by SE emission includes not only inner-shell excitation localized at atoms but also plasmon excitation that is delocalized, the effective inelastic scattering potential in SE imaging becomes larger than the ADF potential [10]. It is noted that the $1{\ }2{\ }\bar 3{\ }0$-type FFT spots appear only in the specific direction indicated by the arrows and not in the direction perpendicular to it. This may be due to the sample vibration and residual 2-fold astigmatism of the condenser lens. The vertical streak accompanied with each FFT spot is caused by the scan noise.

**Fig. 3.**
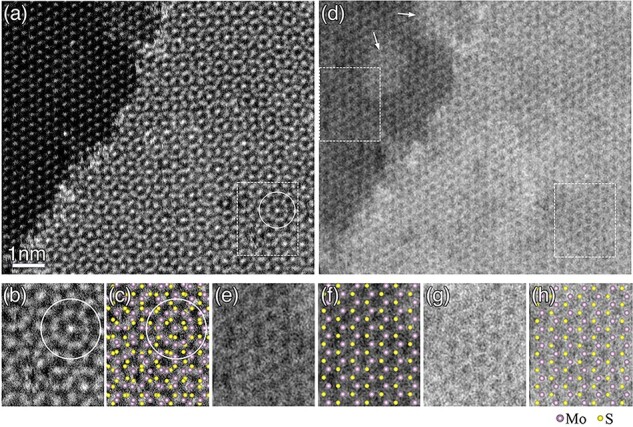
(a) ADF-STEM image of an edge region of a 30°-twisted bilayer of MoS_2_. The slightly darker area in the upper left represents the monolayer region, while the brighter area indicates the bilayer region. The bilayer region shows wheel-shaped arrangements of bright dots, as highlighted by the white circle. (b) Enlarged ADF-STEM image in the area indicated by the white dotted rectangle in (a). (c) Atomic arrangement of the 30°-twisted bilayer of MoS_2_ projected in the [$0{\ }0{\ }0{\ }1$] orientation, overlaid onto (b). The large and small spheres represent Mo and S atoms, respectively. (d) SE image taken simultaneously with the ADF-STEM image of (a). (e) and (g) Enlarged SE images of the areas indicated by the white dotted rectangles on the left and right sides of (d). (f) and (h) Atomic arrangement of the monolayer of MoS_2_ overlaid onto (e) and (g).

Figure 3a shows an ADF-STEM image taken from an edge region of a 30°-twisted bilayer of MoS_2_ at an incidence in the [$0{\ }0{\ }0{\ }1$] orientation. A slightly darker area in the upper left corresponds to the monolayer region, while the other area brighter than the monolayer region corresponds to the bilayer region. The monolayer region shows the same arrangement of bright dots as seen in Fig. 1a. Notably, the bilayer region shows wheel-shaped arrangements of bright dots, as highlighted by a white circle in Fig. 3a.

Figure 3b shows an enlarged ADF-STEM image in the area indicated by the white dotted rectangle in Fig. 3a. Figure 3c shows an atomic arrangement of the 30°-twisted bilayer of MoS_2_ projected in the [$0{\ }0{\ }0{\ }1$] orientation, overlaid onto the ADF-STEM image of Fig. 3c. The wheel-shaped cluster observed in the image is composed of a central bright dot and 12 dots surrounding the center, exhibiting a 12-fold symmetry. This cluster is formed at positions where two Mo atoms from both MoS_2_ layers are projected in close vicinity to each other. The central bright dot of the wheel-shaped cluster corresponds to the superposed two Mo atoms, while the surrounding 12 dots correspond to 12 Mo atoms in the surface and second layers; six Mo atoms are in the surface layer and the other six are in the second layer, and they are alternating. The bright dots observed in the ADF-STEM image show a good agreement to the Mo atom positions in the projected structure of the 30°-twisted bilayer of MoS_2_, indicating that the ADF-STEM image shows the projected structure of the specimen.

Figure 3d shows an SE image acquired simultaneously with the ADF-STEM image of Fig. 3a. Figure 3e and g shows enlarged SE images of the monolayer and bilayer regions indicated by white dotted rectangles on the left and right sides of Fig. 3b, respectively. Figure 3f and h shows the atomic arrangement of monolayer MoS_2_ overlaid onto the SE images in Fig. 3e and g, respectively. Both the monolayer and bilayer show a hexagonal arrangement characteristic of the six-membered ring structure of MoS_2_. This observation suggests that the SE image selectively visualizes one of the two monolayers in the bilayer region. Notably, the bilayer region appears overall brighter than the monolayer region, with the average SE intensity in the bilayer region being 1.72 ± 0.14 times higher than that in the monolayer region. This difference indicates that the net SE yield from the bilayer region is 1.72 times higher than that from the monolayer region overall. It is noted that the SE images show some small patches brighter than the other areas as indicated by the arrows in Fig. 3d, which are not clearly observed in the ADF-STEM image in Fig. 3a. These small patches are considered as very thin residual polymers present on the surface. The SE yield ratio of 1.72 between the monolayer and bilayer regions is calculated using the SE image intensities obtained from areas free from such residual polymer.

**Fig. 4.**
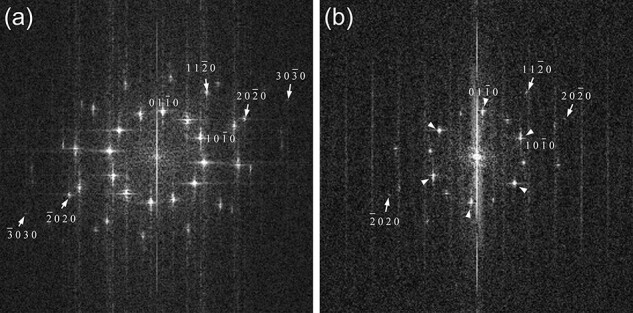
FFT patterns of the ADF-STEM (a) and SE (b) images of the bilayer of MoS_2_. The FFT patterns are interpreted as overlaps of two 6-fold patterns rotated by 30° from each other, with each 6-fold pattern originating from one of the monolayers in the MoS_2_ bilayer. The 12 spots of the $1{\ }0{\ }\bar 1{\ }0$ type in (a) have equivalent intensities, whereas the 12 $1{\ }0{\ }\bar 1{\ }0$-type spots in (b) show six intense ones (large arrowheads) alternating with six weak ones (small arrowheads), exhibiting a 6-fold symmetry.

Figure 4a and b shows typical FFT patterns of the ADF-STEM and SE images of the bilayer region, respectively. The FFT pattern of the ADF-STEM image shows 12 $1{\ }0{\ }\bar 1{\ }0$-type spots with equivalent intensity, exhibiting a 12-fold symmetry. This symmetry is interpreted as an overlap of two 6-fold patterns rotated by 30° from each other, where each 6-fold pattern arises from one of the monolayers in the MoS_2_ bilayer. The 12-fold symmetry indicates that both the surface and second layers of the bilayer are equally visualized by ADF-STEM.

The FFT pattern of the SE image of the bilayer region shows 12 $1{\ }0{\ }\bar 1{\ }0$-type spots similar to the FFT pattern of the ADF-STEM image. However, these 12 spots do not have equal intensities; six intense spots indicated by large arrowheads alternate with six weak spots indicated by small arrowheads, resulting in a 6-fold symmetry. This difference in intensity corresponds to the fact that the SE image visualizes one of the monolayers more intensely than the other. The amplitudes of the six weak spots of the $1{\ }0{\ }\bar 1{\ }0$ type are weaker than the six intense spots by a factor of 0.38 ± 0.09, indicating that the second layer is visualized with 38% contrast of the surface layer. This contrast difference reflects the difference in SE yield, implying that the SE yield when the primary electron is injected into atoms in the second layer is 38% of the SE yield when it is injected into atoms in the surface layer.

**Fig. 5.**
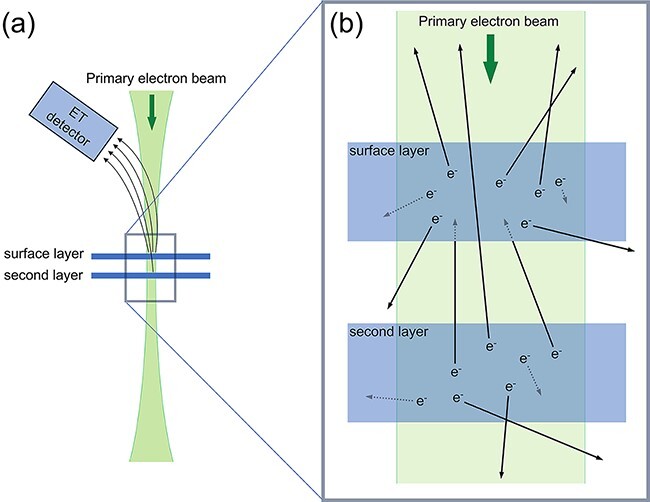
Schematic diagram of the SE generation and emission from the bilayer MoS_2_. (a) The beam illuminates the surface and second layers with the same current density as the focal length of the beam is ∼6 nm, and thus, the amounts of primary SEs generated are also the same. (b) The SEs generated from surface and second layers migrate, and some of the SEs are emitted from the layers. The SEs emitted from the surface layers, positioned on the detector side, can directly reach the detector through free space without encountering potential barriers. Conversely, SEs emitted from the second layers must traverse the surface layer to reach the detector. Some of the SEs from the second layers cannot pass through the surface layer, leading to reduced contrast in the SE images.

Here, we consider the reasons behind the selective visualization of only one of the two layers of MoS_2_ in SE imaging. Figure 5a and b shows schematic diagrams of the illumination of the primary electron beam to the bilayer MoS_2_ and SE emission from the bilayer MoS_2_ sample, respectively. The theoretical focal depth of the incident electron probe, calculated as *λ*/*α*^2^ from the wavelength *λ* and convergence semi-angle *α*, is 6.3 nm, which is significantly larger than the thickness of the MoS_2_ bilayer (Fig. 5a). Given the high energy of the incident electron beam compared to the electrostatic potential of the MoS_2_ layer, the variation of the incident beam current due to the MoS_2_ monolayer can be disregarded. The variation in the electron probe current propagating through the sample was calculated using a multislice calculation in which the thermal vibration of atoms is introduced by a frozen phonon model. The calculation shows that the variation of the 200 keV electron beam passing through a few monolayers of MoS_2_ is <1%, regardless of the probe position. Consequently, both the surface and second layers in the bilayer MoS_2_ receive equal intensity from the incident probe, leading to the assumption that SE generation from these layers is the same. SEs generated in the MoS_2_ layers are shown as ‘e^−^’ in the Fig. 5b.

SEs emitted from the surface layers, positioned on the detector side, can directly reach the detector through free space without encountering potential barriers. Conversely, SEs emitted from the second layers must traverse the surface layer to reach the detector. Consequently, SEs from the second layers are attenuated by the surface layer, leading to reduced contrast in the SE images (Fig. 5b). This phenomenon is akin to Shihommatsu’s findings on graphenes of varying layer numbers deposited on an Ni substrate, which exhibited contrasting SE imaging despite not being at atomic resolution [11]. The contrast variation in the SE image is attributed to SEs emitted from the Ni substrate and attenuated by the graphene layers. Their results show that even a single monolayer of graphene imparts discernible contrast for identifying the number of graphene layers.

The anisotropic angular distribution of fast SEs with an energy of >50 eV generated by primary electrons may also contribute to the difference in the SE yield between the surface and second layers [12]. If there are more fast SEs traveling forward relative to the primary beam than backward, the fast SEs from the surface layer can excite more SEs than those from the second layer, resulting in a more intense visualization of the surface layer. Note that the inelastic mean free path of an electron with an energy of ∼100 eV is ∼0.5–1 nm for most materials [13], which is about the same thickness as that of the bilayer MoS_2_ sample used in this study. Therefore, if fast SEs are generated, they may excite other SEs before escaping from the sample.

The SE intensity in the bilayer region is higher than that in the monolayer region by a factor of 1.72 ± 0.14, indicating a 1.72-fold increase in the net SE yield in the bilayer region. This yield is less than double that of the monolayer region due to the attenuation of SEs emitted from the second layer by the surface layer, as mentioned earlier. FFT analysis reveals that SE images of the surface and second layers are superposed in a ratio of 1 to 0.38 ± 0.09. If the SE emission amount from the monolayer region is the same as that from the surface monolayer of the bilayer region, the total SE emission amount from the bilayer region should be 1.38 times higher than the monolayer region. However, the value is slightly <1.72, the experimental ratio of the SE image intensities of the bilayer region to that of the monolayer region. This suggests that the SE emission per monolayer is slightly higher in the bilayer region than in the monolayer region.

Various excitations such as plasmons, electron–electron interactions and core excitation states induced by primary electrons are known to contribute to SE generation [14]. Among these, surface plasmons contribute more efficiently to SE generation than bulk plasmons or single-electron excitation [15]. The bilayer region may generate more SEs than the monolayer region, as supported by Moyhihan’s electron energy-loss spectroscopy results showing that the bilayer MoS_2_ region excites more plasmons than the monolayer region [16]. Another factor could be the difference in the work function between the monolayer and bilayer. The work function’s dependence on the number of layers was observed in few-layer graphene grown on 6 H–SiC($0{\ }0{\ }0{\ }1$), using photoelectron emission microscopy [17]. Density functional theory and GW calculations also suggest that the work function of the MoS_2_ bilayer is smaller than that of the monolayer, potentially leading to increased SE emission in the bilayer region compared to the monolayer region [18]. However, this discussion assumes that other effects related to SE emission, such as electron density and the electric double layer formed on the surface, are the same for single and double layers of MoS_2_. The influence of the position of the overlapping atoms in the upper and lower layers of MoS_2_ should be investigated in the future.

The spatial resolutions of the SE images in the monolayer and bilayer regions are estimated to be ∼0.16 and 0.14 nm, respectively, derived from the outermost spots of $1{\ }1{\ }\bar 2{\ }0$ and $2{\ }0{\ }\bar 2{\ }0$ observed in the FFT patterns. Notably, the spatial resolution in the bilayer region is comparable to or slightly higher than that in the monolayer region. The high spatial resolution of the SE image of the bilayer region can also be recognized from the fact that the FFT spots from the bilayer region are sharper than those from the monolayer region. The fast SEs emitted from the surface layer may penetrate the second layer and generate additional SEs, some of which escape and contribute to the SE signal, as discussed by Egerton and Zhu [4]. However, the spatial resolution of the SE image is not compromised by the additional SE signal created at different positions from the primary SE because the resolution is determined by the localization of primary-electron scattering. The high spatial resolution of the SE image in the bilayer region can be attributed to several reasons. One possible reason is the higher SE yield from the bilayer region compared to the monolayer region, as spatial resolution is related to the signal-to-noise ratio [14, 19]. Additionally, a higher stability of the bilayer than the monolayer could reduce beam-induced fluctuations in the MoS_2_ structure, contributing to the enhanced spatial resolution observed in the bilayer SE image.

## Conclusion

The current study demonstrates that high-resolution SE imaging can selectively reveal the atomic arrangement of a surface monolayer, even when this monolayer is situated on other materials. These findings highlight the remarkable surface sensitivity of high-resolution SE imaging at the monolayer level. Additionally, our results reveal that the transverse spatial resolution of SE images obtained from the monolayer and bilayer of MoS_2_ does not exhibit significant differences. Further investigations involving high-resolution SE imaging of thicker MoS_2_, where electron channeling effects may occur, are necessary to explore the potential for observing surface structures on different materials. It is also crucial to examine the thickness dependence of the SE yield to determine whether SE production resulting from energy transfer of excited states induced by primary electrons contributes to enhanced SE production beyond volume effects. Such enhancements in SE production could be valuable for low-dose SE imaging. Observation at low acceleration voltages is of extreme importance because it not only suppresses irradiation damage but also increases SE emission. We plan to adjust the optics and perform experimental verification at low acceleration in the future. Future research will be conducted on the formation mechanism of layer-number contrast in SE imaging of atom-layer materials, providing deeper insights into the capabilities and nuances of SE imaging techniques.

## Abbreviations

ADF-STEM, annular dark-field scanning transmission electron microscope; CVD, chemical vapor deposition; SE, secondary electron; STEM: scanning transmission electron microscope.
